# Printable Electrochemical Biosensors: A Focus on Screen-Printed Electrodes and Their Application

**DOI:** 10.3390/s16101761

**Published:** 2016-10-21

**Authors:** Keiichiro Yamanaka, Mun’delanji C. Vestergaard, Eiichi Tamiya

**Affiliations:** 1 Department of Applied Physics, Graduate School of Engineering, Osaka University, Osaka 565-0871, Japan; k-yamanaka@ap.eng.osaka-u.ac.jp (K.Y.); tamiya@ap.eng.osaka-u.ac.jp (E.T.); 2 Department of Food Science and Biotechnology, Kagoshima University, Korimoto, Kagoshima 1-21-24, Japan

**Keywords:** biosensors, electrochemical, point-of-use, screen-printed electrodes (SPEs), disposable electrochemical printed (DEP) chips

## Abstract

In this review we present electrochemical biosensor developments, focusing on screen-printed electrodes (SPEs) and their applications. In particular, we discuss how SPEs enable simple integration, and the portability needed for on-field applications. First, we briefly discuss the general concept of biosensors and quickly move on to electrochemical biosensors. Drawing from research undertaken in this area, we cover the development of electrochemical DNA biosensors in great detail. Through specific examples, we describe the fabrication and surface modification of printed electrodes for sensitive and selective detection of targeted DNA sequences, as well as integration with reverse transcription-polymerase chain reaction (RT-PCR). For a more rounded approach, we also touch on electrochemical immunosensors and enzyme-based biosensors. Last, we present some electrochemical devices specifically developed for use with SPEs, including USB-powered compact mini potentiostat. The coupling demonstrates the practical use of printable electrode technologies for application at point-of-use. Although tremendous advances have indeed been made in this area, a few challenges remain. One of the main challenges is application of these technologies for on-field analysis, which involves complicated sample matrices.

## 1. Introduction

Biosensors were previously defined as devices that respond to chemical species in biological samples or biological components. They are now described as analytical devices composed of a biological recognition element directly interfaced to a signal transducer, which together relate the concentration of a targeted analyte or analytes to a measureable response [1,2]. The principle of a biosensor, therefore, is that a target molecule or a specific event is recognized by a biological molecule. The extent to which the target is recognised, it is detected by a transducer [3]. There are mainly two types of recognition molecules: catalytic- and affinity-based recognition elements. Affinity-based biosensors bind their targets selectively [4]. Examples of affinity-based recognition molecules include signaling receptors, antibodies and nucleic acids. In contrast, catalytic biosensors such as biological cells and enzymes recognize and bind a molecule of interest [4], which then cause some catalyzed chemical conversion of that molecule to a product. The product is subsequently detected by a transducer [5,6]. Classifications of biosensors are often based on the type of recognition molecule and/or the transducer used. There are many different types of transducers, including electrochemical, optical, gravimetric, and piezoelectric devices. A biosensor is described as “an optical biosensor for…”; “an immuno-sensor for…”; “a electrochemical geno-sensor for…”, etc., depending on the transducer and/or recognition element used [4]. Biosensors have achieved and continue to garner interest because of their many attractive features. These include high sensitivity, specificity, ease for integration with other devices, and portability for utilization at-point-of-use [7]. Electrochemical sensors are widely used due to their simplicity, and miniaturization of the device is relatively easy. Moreover, the analysis is rapid, highly sensitive and is of relatively low cost. Consequently, electrochemical transducers are suitable for construction of on-site detection devices. They have seen wide application in many fields including food safety, green energy, bio-medical and environmental monitoring [8,9,10]. One of the challenges that researchers are still striving to address, in all types of biosensors, is the application for real (often complex) samples at the point-of-care and in the field [11].

In order to meet the needs for on-site analysis, it is necessary that we transition away from traditional, commonly used cumbersome beakers and electrochemical cell systems. Nanotechnology and exploitation of new fabrication techniques offer simple, smaller, but robust sensing systems perfectly suited for on-site analyses. Screen-printing technologies have provided just the kind of tool needed to take electrochemical biosensors towards point-of-care. Although not new, having been in existence for over a thousand years [12], it is only in the last two decades or so that screen-printing technology has been exploited in biosensors. To a large extent, nanotechnology has enabled fabrication of printed devices on bendable substrates [13], opening the way for the development of a whole new range of electrode systems. The production of highly reproducible, disposable, stable and relatively low-cost SPEs, has had profound impact on electrochemical biosensor development. Screen-printing technology typically uses stencils, ink and a squeeze blade. It is one of the leading technologies used in the push from lab to point-of-use, and is already establishing its place in a plethora of sensors. This is in large part due to their innate design of easily modifiable ink. Reference, working and counter electrodes have given SPE an unrivaled power of adaptability and excellent accuracy [14,15]. Furthermore, SPEs have garnered much interest because of the attractive features of carbon: chemically inert, low background currents and wide potential window [14]. Besides carbon, which remains top choice in terms of cost, other metals such as gold have their own advantages. The affinity between thiol moieties and gold, which allows SPEs with gold working electrodes to be easily modified with the formation of self-assembled monolayers, has significantly increased the applicability of gold SPEs in electrochemical biosensors [16].

Fabrication of SPEs often follow a similar idea in which the functional electrodes are made using hard and heat-resistive substrates, and the electrode components are created by printing different inks in successive layers to create a finished product. A final curing process is often necessary to anneal the layers of ink together with the underlying substrate for better stability of the electrode [15]. SPEs can be prepared from many types of substrates including plastic films, ceramics, paper sheets, garments, stretch thin films, and epidermis. SPEs are typified by the fabrication of a three-electrode system on the same substrate. In 2012, Li and colleagues wrote an excellent review on basic developments and applications of SPEs in environmental assays over a period between 2009 and 2012. The review provides basic fabrication principles of the printed electrodes, how they are configured and designed, and hybrid analytical techniques based on SPEs [17]. The authors discussed sensitive detection platforms for a wide range of environmental (i) organic samples, including bacteria, herbicides, antibiotic residues and (ii) inorganics, including heavy metal ions. The fabrication of screen-printed electrode arrays, including an array printed at the bottom of a 96-well plate, offers rapid and simultaneous detection of targeted molecules. Li et al. also highlighted the developments that had been made to tackle some outstanding challenges. The problem of fabricating SPE on non-planar surfaces, was addressed by Wang’s group in 2012. Another notable development in this period was the fabrication of a wearable SPE sensor for real-time monitoring of marine contamination. Screen-printing technology is now well established, is economic and easily incorporated in portable devices [18,19].

Another attractive feature of SPEs is that they can be surface-modified in the same way as conventional electrodes, thus enabling increased sensitivity when using superior electro-catalytic properties of nanoparticles. More, they can be used with redox mediators to enhance the catalysis of targets that may otherwise not be easily analysed due to their poor redox activity [20,21,22,23]. In this review, we narrowly focus on the development of electrochemical biosensors based on nucleic acids and antibodies for geno- and immuno-sensing, respectively. We then provide specific examples on how SPEs have been used in sensor development, mostly drawn from our group, for: (i) fabrication and chemical modification of screen-printed sensor surfaces; (ii) further improvements in order to overcome initial drawbacks and (iii) their integration with micro-fluidic systems. Towards the end, we describe other applications of SPEs that demonstrate the versatility of this technology and their potential to be applied at point-of use.

## 2. Printable Electrochemical DNA Biosensors

### 2.1. Electrochemical DNA Biosensors

The most commonly utilized strategy for electrochemical DNA detection exploits the natural biochemical process of hybridization. The basic principle is that a capture single-stranded DNA (ssDNA) molecule, serving as a molecular recognition element, is immobilized on the electrode sensor surface. After, a sample is introduced to the sensor surface. If the sample contains a complementary sequence to the capture ssDNA, a hybridization event occurs, forming a duplex DNA structure [24,25]. The sensor surface of SPEs can easily be modified in order to increase sensitivity of analysis, making them very attractive. For example, a composite of chitosan/Fe_3_O_4_ nanoparticles-modified SPE, was used to fabricate an HIV-I electrochemical biosensor by capturing a DNA hybridization event. Through surface modification with the chitosan/iron oxide NPs, the detection limit was down to pM level, with good stability and reproducibility [26]. Peptide nucleic acids (PNAs) are also utilised as DNA recognition elements [27,28]. PNA is a potent DNA mimic consisting of -(2-aminoethyl)-glycine units that makes it electrically neutral and stable against proteases and nucleases [29].

There are a few different ways to detect hybridization by electrochemical methods. For example, double-stranded DNA (dsDNA) or ssDNA can be modified with electro-active molecules, metal nanoparticles, labeled enzymes, or inorganic DNA intercalators [30]. Ferrocene is one of the most commonly used redox labels due to its high electrochemical activity, and easy utilization with DNA. In the case of longer stranded DNA detection, a sandwich assay is often used. This assay consists of three components: a ssDNA capture probe immobilized on the electrode surface, a target DNA sequence, and a signaling ssDNA probe whose terminal base is modified with ferrocene. The sandwich form is made by hybridization involving these three DNA strands, and results in increased concentration of ferrocene at the electrode surface. The electrochemical response changes proportionally to the concentration of ferrocene on the electrode surface which corresponds to the amount of target DNA, allowing a quantitative detection [31]. Fluorescein is also a common label to detect target DNA, through DNA hybridization [32]. In metal nanoparticle-based detection, a secondary Au-NP-modified DNA probe hybridizes to the target DNA, which is captured on the electrode surface [33]. Using a PNA-mediated PCR and asymmetric PCR technique, Kerman and colleagues developed an electrochemical DNA biosensor for single-nucleotide polymorphism, incorporating cobalt(III) hexamine, [Co(NH_3_)_6_]^3+^ as the hybridization indicator [34]. Detection of DNA damage due to exposure to a mutagen, was achieved using a DNA probe labeled with a photoredox indicator Ru(bpy)_3_^2+^ [33]. In an enzyme-based detection system, a probe DNA is conjugated to a labeled enzyme. After hybridization and washing the electrode surface, a substrate is introduced. The redox activity of the byproduct of enzymatic reaction (for example, hydrogen peroxide) is measured at the electrode surface after applying voltage to the electrode. Erden and colleagues developed an electrochemical enzyme-based sensor using a multi-channel screen-printed array of electrodes, to detect microRNAs. The electrochemical product α-naphthol, was detected using linear sweep voltammetry at a pencil graphite electrode [35]. Basically, in enzyme-labeled electrochemical DNA biosensors, the redox signal changes in proportion to the amount of byproducts of the enzyme reaction, corresponding to the degree of hybridization [36]. These hybridization-based detection methods are very sensitive. However, complicated and laborious processes such as DNA immobilization on electrode and/or DNA labeling are required. Moreover, they are time-consuming because the hybridization reaction is relatively slow and a washing process is also required.

There are several other methods for electrochemical DNA sensing, especially for detecting amplification of DNA. The strategies can be classified into three groups, derived from main PCR target components [37]. The targets are: (i) pyrophosphate, which is a by-product of DNA synthesis [38,39]; (ii) dNTPs, a form of energy consumed during DNA synthesis [40]; and (iii) dsDNA, a form of DNA generated following PCR. Measurement of generated dsDNA is considered the most reliable method for PCR detection compared to the other two targets. This is because there is a lack of specificity and there is limited sensitivity when using pyrophosphate and dNTPs due to excess amounts of ions and/or dNTPs present as part of the PCR solution [41]. For electrochemical measurements of the generated dsDNA, use of dNTPs modified with electro-active molecules such as ferrocene [42,43] or electro-active DNA intercalating molecules have been reported.

The strategy of using intercalating molecules is relatively simple because it can be detected merely by mixing the intercalating molecules with the DNA in solution. That is, there is no need for immobilization on the electrode surface and/or modification of signaling molecules to nucleic acids. Detailed protocols for intercalator-based electrochemical detection of DNA in solution have been reported [44]. The electrochemical response changes when electro-active molecules intercalate DNA through formation of intercalator-DNA complex, and it is dependent on the amount of amplified DNA. DNA amplification is typically done by PCR. Unfortunately, the choice of electrochemical indicators that can be used for this purpose is limited. This is because, although there are many kinds of DNA-intercalating molecules, the electrochemical signal is very weak with most of the methods. In some cases, although the molecules give good electrochemical signals, their affinity towards DNA is low. The three most effective and widely used electroactive DNA intercalating molecules for detection of DNA amplification are methylene blue [45,46,47], osmium bipyridine-based complex and bisbenzimidazole trihydrochloride (Hoechst 33258) [48,49,50]. Since this electrochemical technique is simple, it is considered a suitable candidate for development of an on-site gene detection device.

**Figure 1:**
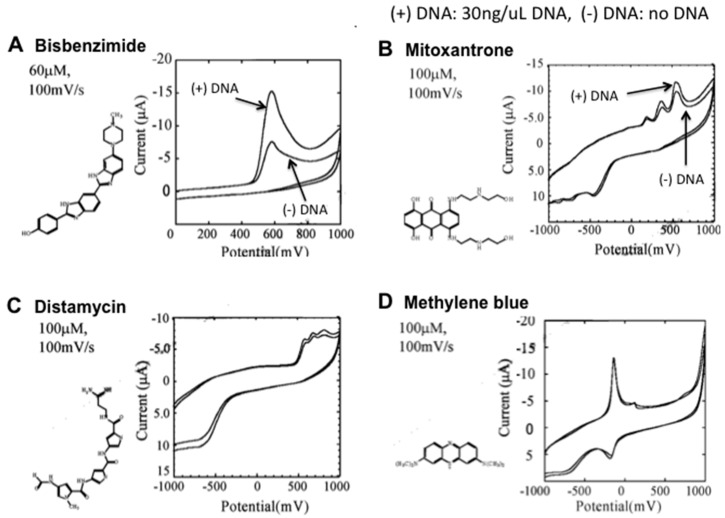
Cyclic voltamograms of various DNA intercalators [51] Hoechst 33258 (**A**); metoxanthrone (**B**); distamycin (**C**); methylene blue (**D**) showing the superiority of Hoescht 33258 as a DNA intercalator, over the other intercalators.

Using disposable electrochemical printed (DEP) chips, we developed a simple intercalator-based DNA biosensor [52]. First, various intercalators (approximately 20) were screened in order to observe the effect of DNA binding on their diffusion coefficients (Figure 1). Changes in the diffusion coefficient are related to formation of complex between DNA and intercalators [51]. The binding of Bisbenzimide (Hoechst 33258) to DNA caused a significant change in its diffusion coefficient.

**Figure 2:**
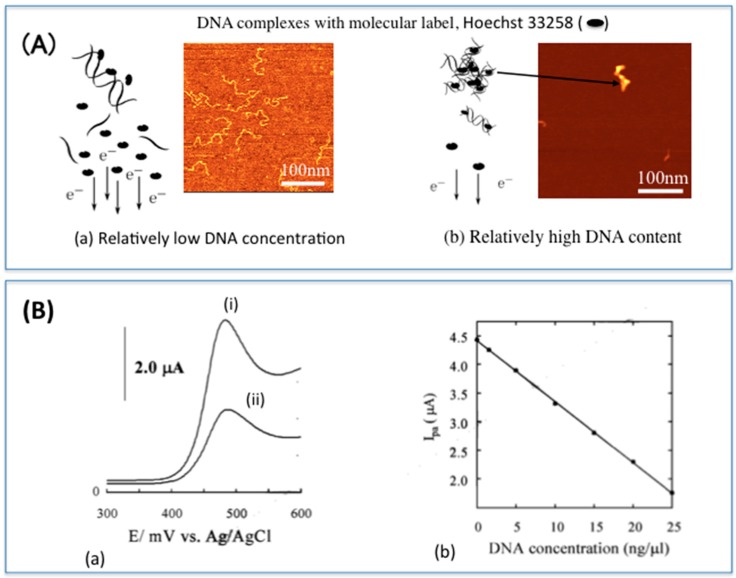
The principle of electrochemical DNA detection using Hoechst 33258 at disposable electrochemical printed (DEP) chips. (**A**) Hoechst 33258 interacts with the DNA resulting in a slow diffusion coefficient of the molecular label to the electrode surface (**a**); When DNA is amplified there is more interaction of DNA with the molecular label and aggregate formation, resulting in even slower diffusion of the molecular label to the electrode surface (**b**); (**B**) Voltammograms showing anodic peak currents of Hoechst 33258 after intercalation (i) with DNA at relatively low concentration; and (ii) amplified DNA. When the DNA is amplified, more Hoechst 33258 intercalates the DNA resulting in higher decrease in current detected (**a**); The degree of interaction is proportional to the current detected (**b**). Part of this figure was used in our original work [52]).

The principle of detection is shown in Figure 2. Basically, free Hoechst 33258 has a high diffusion coefficient and its presence at the electrode surface is shown by an increase in current following a voltammetric analysis. When Hoechst 33258 interacts with DNA, a complex is formed that results in a slower diffusion coefficient of the molecular label, thus lower current is detected. When DNA is amplified, more Hoechst 33258 intercalates the amplified DNA and a further decrease in current is detected. Using this biosensing principle, hepatitis B virus (HBV) from human blood, *Salmonella enteritidis*, and *Streptococcus sobrinus* were detected in less than 2 h. Although interaction of Hoechst 33258 with DNA had been previously reported [53], in our work, we showed that the intercalator caused DNA to aggregate, forming nanostructures that could be clearly observed using atomic force microscopy (AFM) [52]. Of additional interest was how different intercalators induced different DNA nanostructure morphologies (aggregation).

### 2.2. Integration of DNA Biosensors with Microfluidics RT-PCR

Micro-total analysis system (µ-TAS) is an integrated system constructed in a manner that minimizes total processing time, imparts device portability, reduces power consumption and has reagent economy [54,55]. It is typically an integration of several microdevices into a single platform designed to perform a number of tasks including sample preparation, mixing and detection [56,57]. Recently, Cho and colleagues developed a biobarcode assay-based detection of biological agents including botulinum toxin A, with high sensitivity and a response time of 30 min. For detection, they constructed an integrated microdevice comprised of a micropump, a mixer, a separation chamber and an electrophoretic channel. A microfluidic PCR chip was developed as one of the µ-TAS reactor parts for gene detection [58]. In 1998, Kopp et al. presented a continuous-flow microfluidic PCR chip [59]. In this chip, the reaction solution was heated during streaming through a micro-channel by passing over heaters, which were set at optimal temperatures for an efficient PCR process. This way, it was faster compared to conventional thermal cycling devices, which take relatively longer because of the need to control temperature (increase and decrease).

For construction of the detection parts for DNA amplification using microfluidic PCR chips, electrochemical methods have some advantages over commonly used detection methods such as gel electrophoresis and fluorescence observation. These conventional methods require an optical detection apparatus for fluorescence intensity measurement. Although fluorescence-based methods have high sensitivity, the detection device is not amenable to miniaturization. Electrochemical techniques are simple, and the apparatus is relatively easy to miniaturize. Since the electrode design is alterable, it can be adapted to suit a specific device making it suitable as the transducer in an integrated reaction device. Fang et al. developed an integrated microfluidic PCR chip and electrode [46]. They successfully demonstrated real-time measurement of DNA amplification on the chip using sputtered platinum electrodes located in each section of an inner micro-channel. Recently, Fan and colleagues reported on an *intelligent microscale electrochemical device* (iMED) for detection of biomarkers for infectious diseases. This device utilizes a plug-in-cartridge, which is specially adapted for use with SPEs. It is rapid, quantitative, and can simultaneously detect a few different biomarkers [60]. Since SPEs are easily produced en masse and therefore, are of relatively low cost and can be disposable after use, they are very attractive for incorporating in these devices. Disposability is quite important especially for PCR in order to avoid false positives, because DNA amplification methods are very sensitive to contamination. Another approach to integrate microfluidic PCR devices with electrochemical detection devices is by locating the printed electrode near the outlet for end-point measurement of DNA amplification. In such continuous-flow devices, the solution after DNA amplification directly exudes off the outlet of the device, making it easy to integrate with the disposable electrode.

#### Microfluidic RT-PCR Technique for Detection of Influenza Virus

**Figure 3:**
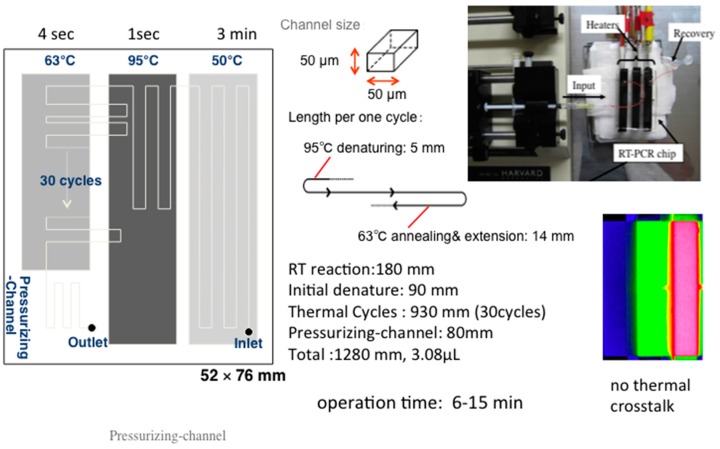
An RT-PCR micro flow chip: Electrochemical semi-real time detection of influenza virus RNA by RT-LAMP on a USB-powered portable potentiostat [46].

To demonstrate application of integrated devices on printed surfaces, here we discuss detection of influenza virus using an integrated microfluidic RT-PCR electrochemical method. The microfluidic device for RT-PCR was newly designed based on the principle discussed in the previous section. Soft lithography techniques were used to fabricate a polydimethylsiloxane (PDMS) microfluidic chip [46]. This RT-PCR chip consisted of four zones: for a RT reaction, an initial denaturation, a thermal cycler zone and a pressurizing channel. The latter was to maintain a set pressure in the inner channel for generation of air bubbles (Figure 3).

Access holes were drilled above inlet and outlet locations. The final step involved an irreversible bonding of a PDMS chip to a glass substrate using oxygen plasma-treatment. Influenza AH1pdm virus RNA was extracted, and used as template RNA. A primer pair for the matrix gene type A-specific sequence, as used for first screening (WHO Information for Laboratory Diagnosis of New Influenza A (H1N1) Virus in Humans) [61] was used. A mixture for continuous-flow RT-PCR contained 20 µM methylene blue (MB) as the redox reporter. The chip was placed on three separate heaters that were maintained at constant temperatures of 50 °C, 95 °C and 63 °C, for denaturation, annealing and extension, respectively, to synthesize cDNA from total RNA molecules.

For electrochemical detection following amplification, MB was utilized as the redox indicator of the amplified DNA. MB, an electro-active molecule with high affinity towards nucleic acids can, through electrostatic interactions, intercalate the double helix [62,63]. Since MB can bind specifically to free guanine bases of ssDNA, it has been used as a reporting element for electrochemical detection of PCR amplification and DNA hybridization [64,65,66].

When DNA is amplified, MB binds to DNA and forms DNA-MB complexes, slowing the diffusion of the DNA-bound reporter molecules compared to free reporter molecules. Thus, following amplification and intercalation, a decrease in current is detected. Moreover, quantitative detection is possible because the degree of reduction in current is proportional to the amount of amplified DNA. In our work, we used disposable SPE chips for electrochemical detection [67,68]. Printed on each single chip were carbon-based working, counter and Ag/AgCl reference electrodes. The surface area of the working electrode was 3.04 mm^2^, and required a volume of approximately 5 μL. PCR solution was allowed to flow freely over the chip surface and was detected using the square wave voltammetry (SWV: frequency, 25 Hz; amplitude, 49.5 mV; scan rate, 48.75 mV·s^−1^; step potential, 1.95 mV). The results showed that specific amplification from influenza A virus was detectable after about 10 min using the microfluidic RT-PCR chip. No amplification was evident for 7 min perhaps due to the flow of the streaming solution being too fast for the biochemical reaction. First, we optimized the sensitivity of the RT-PCR chip, in terms of the number of initial copies required from which detectable amplicons could be obtained. This was done by experimenting with the concentration of the template RNA in the RT-PCR mixture. Specific amplifications were achieved at concentrations >5 copies μL^−1^ of genomic RNA. In electrochemical measurements at SPEs, the mean MB reduction peak currents of samples at ~−0.38 V were decreased to approximately 70%, 62%, 46% of control values. The reduction was proportional to the concentrations of influenza RNA. The results clearly showed that the detection of microfluidic RT-PCR amplification of influenza A RNA was possible using MB-mediated electrochemical measurements within 15 min, at disposable SP carbon electrodes. Interested readers may refer to the original report by Yamanaka and colleagues for more details [46].

## 3. Other Printable Electrochemical Biosensors

### 3.1. Electrochemical Immunosensors

Immunoassay is a widely used technique for protein detection, utilizing the natural affinity of antibodies towards antigens. Immunoassays date as far back as 1959 [69]. In optical-based immunoassays such as enzyme-linked immunosorbent assay (ELISA), primary antibodies capture target proteins. The primary antibodies can be immobilized onto solid surfaces such as glass beads, magnetic beads, plastic plates, etc., followed by sample introduction. If the targeted for antigen is present in the sample, the antigen binds with the immobilized antibody. A secondary antibody, most commonly conjugated with an enzyme is introduced, followed by washing [70,71]. Then a chemiluminescent substrate is added for enzyme reaction, and measured optically to detect and/or quantify the target antigen. Electrochemical immunosensors are developed for applications in various fields including medical, environmental and food. Similar to DNA biosensors, electrochemical immunosensors utilizing SPEs are attractive for on-site analyses because they are relatively affordable, can be portable and are disposable. Vig and colleagues fabricated an impedimetric immunosensor on Au/Ag-modified SPEs, and demonstrated its applicability to detect carcinogenic mycotoxin, aflatoxin M1, in milk [72]. For immunosensing based on the electrochemical methods, immobilization of the recognition element onto the electrode surface is necessary. There are a few different types immobilization procedures including adsorption, entrapment, cross-linking and covalent coupling (random or orientated) [73,74,75,76]. The most widely used surface modification technique is through formation of self-assembled monolayer (SAM) on a gold electrode surface. SAM is normally created by the interaction between the gold surface and a thiol base, via a disulphide bridge, at one end of linker molecule, with functional bases for interacting with an antibody on the other end. The antibody is immobilized on the electrode surface via SAM created with the linker molecules through covalent bonding [75]. An example of this surface chemistry modification was employed by Xu and colleagues. The group developed an electrochemical immunosensor for detection of DNA methylation by assaying DNA methytransferase activity. A complex detection platform was immobilized on electrode surface through SAM formation of AuNPs [77].

#### Gold-Linked Electrochemical Immuno-Assay (GLEIA)

The majority of electrochemical immunoassays have employed enzymatic labels such as alkaline phosphatase, horseradish peroxidase, similar to ELISA. There are alternative strategies that utilize metal nanoparticles (NPs) instead of enzymes, as labels for electrochemical immunosensors [78,79]. Metal NPs have high conductivity and catalytic property, and are more stable than enzymes, making them hugely attractive. Direct fabrication of Au NPs on printed electrode surfaces has been reported [80,81]. A dual sensor for simultaneous detection of free and total prostate specific antigen was developed on SPE surfaces. The authors used AuSPEs, screen-printed carbon electrodes and SPEs modified with nanogold. The latter gave the best performance in terms of sensitivity as well as reproducibility [82].

**Figure 4:**
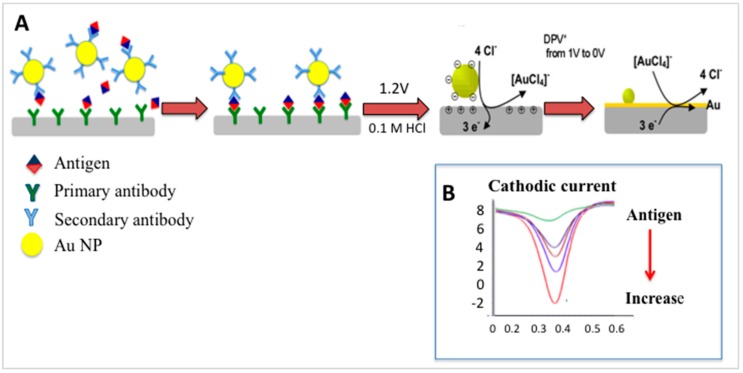
(**A**) Illustration of the principle of gold-linked electrochemical immunoassay (GLEIA). A primary antibody is immobilized directly on an electrode surface, and a typical sandwich-type immunoreaction carried out. The secondary antibody is labeled with Au NPs. In the presence of 0.1 M HCl, and upon application of approximately 1.2 V, the Au NPs are pre-oxidized from Au^0^ to Au^3+^. A differential pulse voltammetry (DPV) from 0 V to 1.0 V immediately after the pre-oxidation step reduces the oxidised NPs back to Au^0^ (this figure is a partial reproduction of our previous figure in Vestergaard et al. [1]); (**B**) A voltammogram of the reduction signal of Au NPs on carbon SPE. The signal increases proportionally to increase in antigen concentration (part of the figure is from our previous work: Idegami et al. [83]).

We now describe an example of a simplified electrochemical-based immunosensing method, gold-linked immune-assay (GLEIA) [83]. In this method, the principle of detection is based on the direct electrochemical activity of Au NPs on SPEs (Figure 4). A primary antibody is immobilized directly on an electrode surface, and a typical sandwich-type immunoreaction carried out. As can be seen in Figure 4, the secondary antibody is labeled with Au NPs. In the presence of 0.1 M HCl, and upon application of approximately 1.2 V, the Au NPs are pre-oxidized from Au^0^ to Au^3+^. A differential pulse voltammetry (DPV) from 0 V to 1.0 V immediately after the pre-oxidation step reduces the oxidised NPs back to Au^0^. The reduction process is detected at approximately 0.48 V. Based on GLEIA, human chorionic gonadotropin hormone could be detected with very high sensitivity [83,84].

### 3.2. Electrochemical Enzyme-Based Biosensors

Enzyme-based biosensors were the first type of biosensors to be developed. Clark developed the first biosensor for detection of glucose in 1962 [85], opening the way to this now huge important field of biosensing. There have since been much research leading to development of three generations of glucose biosensors, and of course, the glucose sensors for use at-point-of-care [86,87]. Enzymes are still perhaps the most utilized recognition elements for biosensor development, with wide applications in numerous fields. Ispas and colleagues wrote a review paper on developments in enzyme-based biosensors for biomedical analysis [88]. This review, focused on design, performance and applications, including detection at SPEs, is an excellent reference for those particularly interested in enzyme-based biosensors, and how they have advanced over time, including the use of printable technology. Perhaps due to their fast response and specificity towards their substrates, enzymes continue to play a huge role in biosensor design. In 2010, Dounin et al. developed an enzyme biosensor for the sensitive detection of paraoxon (LoD: 10 ppt) and carbonfura (LoD: 10 ppb) by monitoring their inhibitive ability on acetylcholine esterase activity [89]. Alonso-Lomillo’s group have developed electrochemical enzyme-based biosensors, targeting a range of important molecules including toxins, drugs, and biogenic amines, at SPEs [90,91,92]. Screen-printed amperometric enzyme biosensors, surface-modified for increased sensitivity as described in previous sections, have been widely reported [93]. Our group developed a compost maturity sensor utilizing three parameters: pH, NH_4_^+^ concentration, and phosphatase activity present in water extracts of compost samples [94]. Apparent phosphatase activity in crude test solutions was determined using DPV at screen-printed carbon strips coated with α-naphthyl phosphate (α-NP) in Nafion film. Alkaline phosphatase (ALP) in Tris-HCl buffer and acid phosphatase (ACP) in citric acid buffer were used to validate the developed sensor. An aqueous extract of the compost sample, spiked with enzymes, was analyzed and showed a change in anodic current at peak potential of the product, α-naphthol. Water extracts of compost samples of various ages were analyzed using the developed compost maturity sensor system, and conventional germination tests. Using multiple regression analysis, a germination index (GI) was established using a multi-linear regression equation consisting of pH, NH_4_^+^ concentration, and the phosphatase activity. The calculated GI having a good correlation with the measured GI of the corresponding compost samples provided an equation for determining compost stability using our portable sensor system in a simple and quick manner, on-site. Enzyme biosensors are equally flexible, and robust, allowing exploitation of nanomaterials including carbon nanotubes, metal NPs, metal oxide NPs and composite materials, at SPEs for fabrication of more sensitive enzyme-based electrochemical biosensors [95,96].

Printed electrochemical sensors have also been applied for detection of environmental pollutants including organic and inorganic molecules [97,98]. Long’s group developed a sensor for multiple detection of heavy metals at SPE coated with multi-walled carbon nanotubes followed by modification with mercury nanodrops [99]. The developed sensor was able to detect cadmium, lead and copper ions in river samples. In the next section, we briefly present a technology that has been developed to allow integration and portability for on-site measurement. It is used in conjunction with SPEs.

## 4. Miniaturized Printed and Portable Electrochemical Biosensors

**Figure 5:**
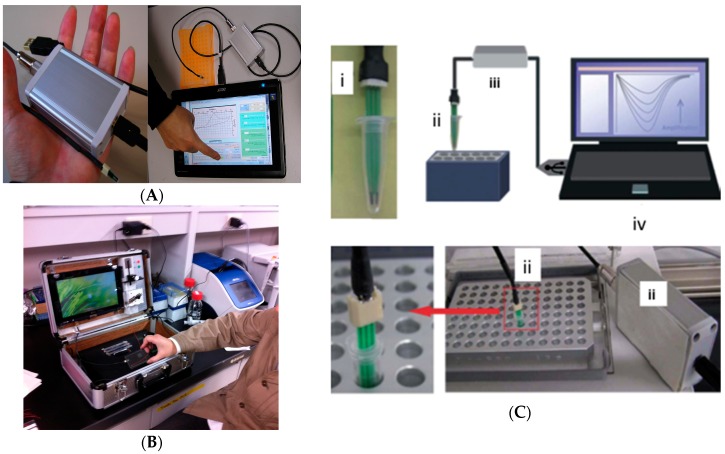
Portable electrochemical devices used with disposable electrochemical printed (DEP) chips: Handheld potentiostat, operated by a simple PC or tablet (**A**); conveniently fitted in a small portable case (**B**); The DEP chips can conveniently fit into PCR tubes (i) for DNA amplification (ii) and subsequent detection (iii, iv) (**C**); Part of this figure was used in our original work [52].

A lot has been reported on the advantages of electrochemical biosensors including ease of integration, and miniaturization leading to portability for application at point of need/use and affordability. In this short section, we provide an example of the afore-said advantages of electrochemical biosensors, we briefly show some of the devices that have been developed to support portability (Figure 5).

## 5. Future Outlook and Concluding Remarks

Electrochemical biosensors have come a long way since the first one was reported by Clark [85] in 1962. Many challenges still remain, with application at points-of-need/use being one of the major ones. In order to be able to effectively conduct on-site analysis, it is necessary to change the analysis systems to smaller, portable, and disposable tools. That is, transition away from the traditional cumbersome beaker-type electrochemical cells and bulky electrodes. The development of screen-printed technologies has gone a long way to addressing this issue because it enables easy integration, improves portability, and the electrode systems used are disposable. The latter provides simplicity and drastically reduces contamination. Screen-printing allows for reproducible electrode surfaces to be made en-masse, which would normally require prior modification of the electrode surface—as in the case of drop-casting techniques. Being able to customize the working electrode, tailored to need, is another advantage offered by screen-printing [15]. More, mass fabrication makes them relatively cheap. SPEs are very versatile in many ways, including (i) ways in which SPEs can be modified, through surface chemistry modification and/or by directly modifying the composition of the ink; (ii) incorporation of biomaterials with natural specificity for target molecules, and materials that aid in increasing sensitivity [100]. On the latter point, we have seen exploitation of transition metals, metal oxides, carbon-based nanoparticles and composites used to increase surface area for sensing in order to increase electron transfer process [15,101]. Wang’s group developed AuSPEs, modified with a ternary SAM monolayer of hexanedithiol, specific thiolated capture probes, and 6-mercapto-1 hexanol. Using a labeled capture probe, target DNA in raw biological samples, could be detected at high sensitivity (pM concentration) [102]. The versatility of SPEs was reported recently by Baraket and colleagues who fabricated a fully integrated immuno-based detection platform [103]. The authors demonstrated the potential of the biosensor towards multiple detection of cytokines at sensitivities in the pg/mL levels.

Nanomaterials have also been used as carrier beacons for indirect, yet highly robust and accurate means for detecting target molecules [46]. With the discovery of new nanomaterials, we expect researchers to quickly exploit them for development of even better sensing methods and devices, as well as explore new ways to advance screen-printing technologies. The use of biological as well as bio-mimetic materials is likely to increase with increased understanding of their interactions and mechanisms. The commercialization of SPEs pre-modified with stable sensitivity-enhancing molecules and/or molecular labels such as Au NPs, will substantially reduce sensor-fabrication time [100], and also allow wide application by researchers less experienced in device fabrication. We have also shown in this review, a small potentiostat that can be powered by UBS connected to a simple notepad, conveniently designed to fit SPEs for on-field use. Although the issue of sensitivity, selectivity and interference when handling biological samples or other complex sample matrices, is still to be resolved, there has been some successes that offer promise for easy and rapid real-sample application in the fore-seeable future. Screen-printing technology has enabled the development of bandage-based wearable potentiometric sensor fabricated using SP technology for measuring wound pH [104], and wearable electrochemical biosensors, in various forms including tattoos and patches for use at point-of-care [105,106]. Non-complicated integration with smart devices and wire-less technology would allow easy and rapid monitoring of these patients wearing these point-of-care biosensors, remotely.
